# Role of Diffusion Weighted Imaging and Contrast-Enhanced MRI in the Evaluation of Intrapelvic Recurrence of Gynecological Malignant Tumor

**DOI:** 10.1371/journal.pone.0117411

**Published:** 2015-01-28

**Authors:** Kazuhiro Kitajima, Utaru Tanaka, Yoshiko Ueno, Tetsuo Maeda, Yuko Suenaga, Satoru Takahashi, Masashi Deguchi, Yoshiya Miyahara, Yasuhiko Ebina, Hideto Yamada, Masakatsu Tsurusaki, Yukihisa Tamaki, Kazuro Sugimura

**Affiliations:** 1 Department of Radiology of Kobe University Graduate School of Medicine, Kobe, Japan; 2 Department of Obstetrics and Gynecology of Kobe University Graduate School of Medicine, Kobe, Japan; 3 Department of Diagnostic Radiology, Kinki University School of Medicine, Osaka, Japan; 4 Department of Radiation Oncology, Shimane University School of Medicine, Shimane, Japan; University of Munich, GERMANY

## Abstract

**Background and Purpose:**

To investigate the diagnostic performance of diffusion-weighted imaging (DWI) and contrast-enhanced imaging in combination with T2-weighted imaging (T2WI) for magnetic resonance imaging (MRI) evaluation of intrapelvic recurrence of gynecological malignancies.

**Materials and Methods:**

Sixty-two patients with suspected intrapelvic recurrence of gynecological malignancies underwent pelvic MRI including T2WI DWI, and contrast-enhanced imaging. Diagnostic performance for detection of local recurrence, pelvic lymph node and bone metastases, and peritoneal lesions was evaluated by consensus reading of two experienced radiologists using a 5-point scoring system, and compared among T2WI with unenhanced T1-weighted imaging (T1WI) (protocol A), a combination of protocol A and DWI (protocol B), and a combination of protocol B and contrast-enhanced imaging (protocol C). Final diagnoses were obtained by histopathological examinations, radiological imaging and clinical follow-up for at least 6 months. Receiver operating characteristic (ROC) analysis and McNemar test were employed for statistical analysis.

**Results:**

Locally recurrent disease, lymph node recurrence, peritoneal dissemination and bone metastases were present in 48.4%, 29.0%, 16.1%, and 6.5% of the patients, respectively. The patient-based sensitivity, specificity, accuracy, and area under the ROC curve (AUC) for detection of intrapelvic recurrence were 55.0, 81.8, 64.5% and 0.753 for protocol A, 80.0, 77.3, 79.0% and 0.838 for protocol B, and 80.0, 90.9, 83.9% and 0.862 for protocol C, respectively. The sensitivity, accuracy, and AUC were significantly better for protocols B and C than for protocol A (p<0.001). There was no significant difference between protocols B and C.

**Conclusion:**

MRI using a combination of DWI and T2WI gives comparatively acceptable results for assessment of intrapelvic recurrence of gynecological malignancies.

## Introduction

For patients with gynecological malignancies, early, non-invasive and accurate assessment of recurrence is crucial in order to decide whether salvage treatment or palliation is appropriate, thus optimizing not only survival and quality of life, but also resource allocation. It is desirable to identify recurrences before symptoms develop, as survival decreases once patients become symptomatic. Although the majority of recurrences are observed within the pelvis, early detection may be diagnostically challenging. The symptoms can be non-specific, and physical examination of the irradiated pelvis is often limited. For monitoring of patients after treatment for gynecological malignancies, the mainstay is analysis of clinical and biochemical (tumor marker) parameters combined with computed tomography (CT) surveillance. Magnetic resonance imaging (MRI) is usually reserved for problem-solving to clarify the nature of indeterminate lesions, most commonly in the pelvis. Contrast-enhanced MRI, especially dynamic contrast-enhanced MRI, has been assessed as a tool for detection of tumor recurrence and distinguishing it from post-surgical and post-radiotherapy changes [1–5].

Recent technical advances in diffusion-weighted imaging (DWI) have greatly enhanced the clinical value of MRI. DWI can provide excellent tissue contrast based on molecular diffusion, and may be able to demonstrate malignant tumors. In combination with conventional MRI, DWI and apparent diffusion coefficient mapping can provide additional information in patients with gynecological malignancies, identifying additional sites of pelvic tumors and improving the degree of confidence in image interpretation [6–9]. Only one previous study has demonstrated the usefulness of fused DWI and T2-weighted imaging (T2WI) for depicting local tumor recurrence in patients with primary pelvic malignancies [10]. The clinical usefulness of DWI for evaluation of locally recurrent gynecological malignancies needs to be clarified. If the performance of DWI were to equal that of contrast-enhanced imaging for detection of recurrent gynecological malignancies, then administration of contrast medium might become unnecessary. In the present study, we evaluated the diagnostic ability of DWI and CEI in combination with T2WI and unenhanced T1-weighted imaging (T1WI) and assessed the clinical utility of DWI for evaluation of intrapelvic recurrence of gynecological malignant tumors.

## Materials and Methods

This retrospective study was performed in accordance with the principles of the Declaration of Helsinki. The institutional review board (Kobe University Hospital, Japan) approved this retrospective study (No 1469) and actually waived the need for patient informed consent.

### Patients

Between 2009 and 2013, 80 patients in whom intrapelvic recurrence of gynecological malignancies was suspected on the basis of elevated tumor marker levels, abnormal findings of CT, MRI, or ^18^F-fluorodeoxyglucose positron emission tomography/computed tomography (FDG-PET/CT), or an abnormal Papanicolaou smear after resection of the primary tumor underwent pelvic MR studies for further evaluation. Eighteen patients were excluded because they did not receive intravenous administration of contrast media at the time of MRI scan. The final population comprised 62 eligible patients with a mean age of 56.9 years (range, 28–83 years). Therapies for the primary pelvic malignant tumor included surgery alone (*n =* 34), surgery plus chemotherapy (*n =* 17), surgery plus radiotherapy (*n =* 7), and surgery plus chemoradiotherapy (*n =* 4). In the 34 patients who underwent surgery only, the MRI studies were performed between 6 and 72 months (median 19 months) afterwards. In the 28 patients who underwent surgery plus chemotherapy and/or radiotherapy, the MRI studies were performed between 3 and 24 months (median 8.5 months) after completion of the chemotherapy or radiotherapy. The primary tumors were carcinomas of the cervix (n = 21), ovary (n = 20), endometrium (n = 14), vagina (n = 2) and vulva (n = 1), and uterine sarcoma (n = 4).

### MRI

MRI was performed using a 1.5-T system (n = 43) (1.5T Gyroscan Intera NT or Achiva 1.5T Nova dual; Philips Medical Systems, Best, The Netherlands) or a 3-T system (n = 19) (Achieva; Philips Medical Systems, Best, The Netherlands) using a body coil for excitation and a pelvic phased-array coil for signal reception. The MRI parameters varied throughout the time of MRI data acquisition owing to adaptations of our standard clinical protocol. Peristalsis was suppressed with intramuscular administration of 20 mg of scopolamine butylbromide (Buscopan; Boehringer Ingelheim, Yamagata, Japan) or 1 mg of glucagon (Glucagon-G Novo; Eisai Co. Ltd., Tokyo, Japan). Axial and sagittal fast-spin-echo T2WIs were acquired with a repetition time (TR)/echo time (TE) of 3300–4800 ms/90–100 ms, a 4–5 mm slice thickness/1 mm gap, a 20- to 24-cm field of view (FOV), and a 192×256–256×256 matrix. Unenhanced T1WIs were acquired in the axial and sagittal planes with a spin-echo TR/TE of 525–700/6–10 ms, a 4–5-mm slice thickness/1 mm gap, a 20- to 24-cm FOV, and a 192×256–256×256 matrix. Axial DWI was obtained along three orthogonal directions using spin-echo-type single-shot echo planar imaging with the following parameters: b value = 0 and 1000 ms/mm^2^, TR/TE = 3000–4000/60–68 ms, a 3–4 mm slice thickness/no gap, a 24- to 45-cm FOV, and a 102×128–128×192 matrix. In half of the 62 patients (n = 31), after 0.1 mmol/kg gadolinium diethylenetriaminepentaacetic acid (Magnevist; Bayer Schering Pharma, Osaka, Japan) had been administered at a rate of 2.0–3.0 ml/s, followed by a saline flush (15 ml at 2.0–3.0 ml/s), multiphase dynamic images (a 20-seconds acquisition time, 9 sections) were obtained using three-dimensional, fast-gradient-echo, T1-weighted fat-suppressed, sagittal or axial sequences (TR/TE = 14–15/7–8 ms, a 2–3 mm slice thickness/no gap, a 28- to 40-cm FOV, and a 128×128–192×256 matrix). Finally, we also obtained delayed (4–5 min later) T1-weighted fat-suppressed axial and sagittal sequences sequentially, with parameters similar to those used before injection of gadolinium diethylenetriaminepentaacetic acid. In the other 31 patients, after a single injection of gadolinium diethylenetriaminepentaacetic acid at a dose of 0.1 mmol/kg body weight, we obtained T1-weighted fat-suppressed axial, sagittal, and coronal sequences sequentially, using parameters similar to those used before injection of gadolinium diethylenetriaminepentaacetic acid.

### Image analysis

Two radiologists with 13 and 6 years of gynecological MRI experience, who had no knowledge of either the histologic findings or the clinical data, retrospectively evaluated the three protocols for the following findings: (a) local pelvic recurrence (hysterectomy scar, vagina, ovariectomy scar), (b) pelvic lymph node metastasis, (c) intrapelvic peritoneal dissemination, and (d) pelvic bone metastasis using a five-point grading system (1: definitely absent, 2: probably absent, 3: indeterminate, 4: probably present, and 5: definitely present). The data for protocol A was that obtained by T2WI and unenhanced T1WI, data for protocol B consisted of the data from protocol A and DWI, and the data for protocol C consisted of the data from protocol B and contrast-enhanced imaging. Each dataset was reviewed with the consensus of the two readers after a minimum interval of 3 weeks to avoid any decision threshold bias due to reading-order effects.

We referred to several previous standard criteria for restaging of gynecological malignancy on the basis of MRI interpretation [1–10]. Recurrent disease (local recurrence, peritoneal dissemination and bone metastasis) appears as a mass of intermediate to high signal intensity on T2WIs and low signal intensity on T1WIs. Areas of tumor recurrence appear bright on DWIs and demonstrate low signal intensity on apparent diffusion coefficient maps. A focal lesion with relatively early enhancement (45–90 s) on dynamic contrast-enhanced imaging or a different enhancement pattern relative to the surrounding tissue in a delayed scan was judged to be a recurrent lesion. Lymph node swelling larger than 1 cm in short-axis diameter was graded as metastasis. Furthermore, the presence of a central unenhanced area suggesting central necrosis was considered to be a malignant sign, whereas the presence of peripheral low attenuation suggesting a fatty hilum within a lymph node was considered to be a benign sign.

### Standard of reference

Tumor recurrence was confirmed by surgery, percutaneous biopsy and by monitoring the progression or remission of the disease using clinical examinations, assessment of serum tumor marker levels, and imaging (CT, MRI, and FDG-PET/CT) during and after medical therapy.

For the patients who underwent surgery, the surgical and pathologic reports and the clinical charts were examined to determine the presence or absence of lesions. For the patients who did not undergo surgery, the following imaging criteria were applied as the standard of reference: lesion found at follow-up examination after the initial examination; lesion larger than that apparent during the initial examination; and lesion smaller in the follow-up examination after chemotherapy or radiotherapy than during the initial examination. If the lesions detected at the initial examination had resolved within three months without therapy, and no lesion had developed in a patient during the six months after the initial examination, we considered the lesion to be false positive and categorized the case as lesion absent. When the follow-up examination showed no lesion, an additional examination performed more than six months after the initial one was required in order to categorize the case as lesion absent.

### Statistical analysis

To estimate the utility of each imaging modality for diagnosis of intrapelvic recurrence, receiver operating characteristic (ROC) analysis was used on a per-patient basis. Differences in assessment between the three sets of the area under the ROC curve (AUC) were tested for significance by Cochran Q test. Any two sets of AUC were tested using McNemar’s test with Bonferroni adjustment. Tests for differences in sensitivity, specificity, and accuracy between imaging protocols were conducted using the McNemar test. To calculate the sensitivity and specificity of each modality, scores of 4 and 5 were considered positive. In all analyses, a P value of less than 0.05 was considered to indicate a statistically significant difference. Statistical analysis was performed using the SAS software package (version 9.3; SAS Institute).

## Results

**Fig 1:**
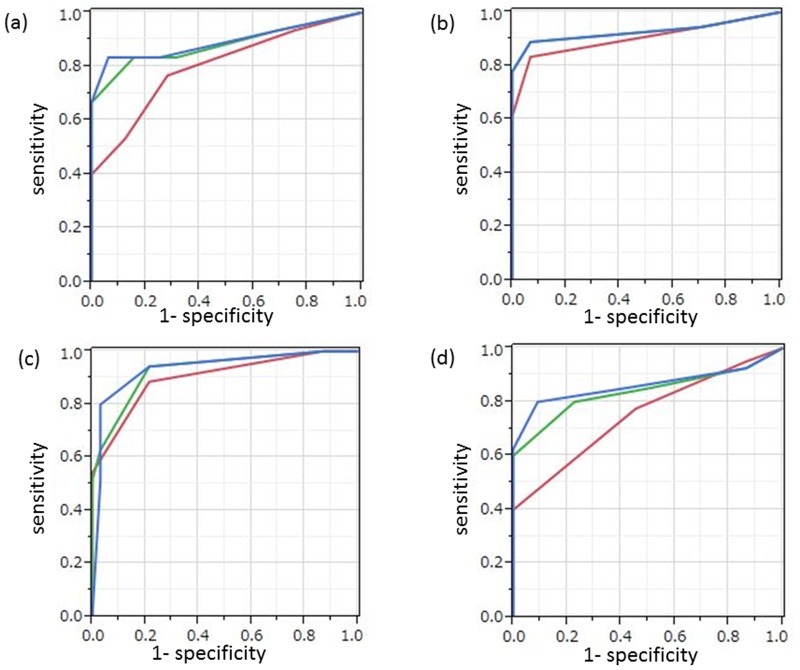
ROC curves for protocol A (red), protocol B (green), and protocol C (blue) in depicting (a) local recurrence, (b) intrapelvic lymph node recurrence, (c) intrapelvic peritoneal dissemination, and (d) intrapelvic recurrence on a per-patient basis. (a) The AUCs calculated for protocol B (0.881) and protocol C (0.892) were significantly higher than that for protocol A (0.800). (b) The AUC calculated for protocols B and C (0.927) was not significantly higher than that for protocol A (0.902). (c) The AUCs calculated for protocol B (0.924) and protocol C (0.931) were significantly higher than that for protocol A (0.885). (d) The AUCs calculated for protocol B (0.838) and protocol C (0.862) were significantly higher than that for protocol A (0.753).

Documented positive locally recurrent disease, pelvic lymph node recurrence, peritoneal dissemination and bone metastases were present in 48.4% (30/62), 29.0% (18/62), 16.1% (10/62), and 6.5% (4/62) of the patients, respectively. Patient-based sensitivity, specificity, accuracy, and AUC for depicting local recurrence, pelvic lymph node metastasis, intrapelvic peritoneal dissemination, pelvic bone metastasis, and overall (intrapelvic) recurrence for the three protocols are shown in Table 1 and Fig. 1.

**Table 1:** Comparison of three protocols for diagnosing intra-pelvic recurrence on a per patient basis.

	Sensitivity	Specificity	Accuracy	AUC
	95% CI	95% CI	95% CI	95% CI
Local recurrence				
Protocol A	53.3% (16/30)* **	87.5% (28/32)	71.0% (44/62)	0.800^#^ **
	35.5–71.1	76.1–98.9	59.9–82.3	0.670–0.888
Protocol B	83.3% (25/30)*	84.4% (27/32)	83.9% (52/62)	0.881^#^
	69.9–96.6	71.8–97.0	74.7–93.1	0.754–0.947
Protocol C	83.3% (25/30)**	93.8% (30/30)	93.3% (28/30)	0.892**
	69.9–96.6	85.5–100	80.8–96.6	0.764–0.954
Pelvic lymph node metastasis				
Protocol A	61.1% (11/18)	97.7% (43/44)	87.1% (54/62)	0.902
	38.6–83.6	93.3–100	78.8–91.4	0.737–0.968
Protocol B	77.8% (14/18)	97.7% (43/44)	91.9% (57/62)	0.927
	58.6–88.1	93.3–100	85.1–98.7	0.744–0.983
Protocol C	77.8% (14/18)	97.7% (43/44)	91.9% (57/62)	0.927
	58.6–88.1	93.3–100	85.1–98.7	0.744–0.983
Peritoneal dissemination				
Protocol A	40.0% (4/10)	100% (52/52)	90.3% (56/62)	0.885
	9.6–70.4		82.9–97.7	0.716–0.959
Protocol B	50.0% (5/11)	96.2% (50/52)	88.7% (55/62)	0.924
	19.0–81.0	91.0–100	80.8–96.6	0.776–0.977
Protocol C	70.0% (7/10)	96.2% (50/52)	91.9% (57/62)	0.931
	41.6–98.4	94.6–100	85.1–98.7	0.772–0.982
Bone metastasis				
Protocol A	100% (4/4)	100% (58/58)	100% (62/62)	1.000
				
Protocol B	100% (4/4)	100% (58/58)	100% (62/62)	1.000
				
Protocol C	100% (4/4)	100% (58/58)	100% (62/62)	1.000
				
Overall (Intrapelvic recurrence)				
Protocol A	55.0% (22/40)* **	81.8% (18/22)	64.5% (40/62)	0.753* **
	39.6–70.4	65.7–97.9	52.6–76.4	0.622–0.850
Protocol B	80.0% (32/40)*	77.3% (17/22)	79.0% (49/62)	0.838*
	67.6–92.4	68.4–86.2	68.9–89.1	0.716–0.914
Protocol C	80.0% (32/40)**	90.9% (20/22)	83.9% (52/60)	0.862**
	67.6–92.4	78.9–100	74.9–95.0	0.741–0.932

AUC: area under the receiver operating characteristic analysis

CI: confidence interval

Protocol A: T2-weighted imaging (T2WI) and unenhanced T1-weighted imaging (T1WI)

Protocol B: Combination T2WI plus unenhanced T1WI and diffusion weighted imaging (DWI)

Protocol C: Combination of T2WI plus unenhanced T1WI, DWI and contrast-enhanced imaging

*, **: statistically significant different (p<0.001)

^#^: statistically significant different (p<0.05)

On the diagnostic performance of intrapelvic local recurrence, the sensitivity, accuracy, and AUC for protocol B were significantly better than those for protocol A (p = 0.0077, p = 0.013, and p = 0.0044, respectively). The sensitivity, accuracy, and AUC for protocol C were significantly better than those for protocol A (p = 0.0077, p = 0.0026, and p = 0.0043, respectively). Although the specificity, accuracy, and AUC for protocol C were better than those for protocol B, no significant difference was observed (p = 0.25, p = 0.25, and p = 0.62, respectively).

On the diagnostic performance of pelvic lymph node recurrence, although the sensitivity, accuracy, and AUC for protocol B or C were better than those for protocol A, no significant difference was observed (p = 0.25, p = 0.25, and p = 0.25, respectively).

On a per patient basis, the sensitivity, specificity, accuracy, and AUC for detection of intrapelvic peritoneal dissemination were 36.4%, 100%, 88.7%, and 0.895 for protocol A, 54.5%, 98.0%, 90.3%, and 0.939 for protocol B, and 72.7%, 98.0%, 93.5%, and 0.950 for protocol C, respectively. On the diagnostic performance of intrapelvic peritoneal dissemination, although the sensitivity, accuracy, and AUC for protocol B were better than those for protocol A, no significant difference was observed (p = 0.93, p = 0.48, and p = 0.29, respectively). Although the sensitivity, accuracy, and AUC for protocol C were better than those for protocol B, no significant difference was observed (p = 0.25, p = 0.29, and p = 0.54, respectively).

On the diagnostic performance of intrapelvic recurrence, the sensitivity, accuracy, and AUC for protocol B were significantly better than those for protocol A (p = 0.0044, p = 0.0077, and p = 0.0097, respectively). The sensitivity, accuracy, and AUC for protocol C were significantly better than those for protocol A (p = 0.0044, p = 0.0015, and p = 0.0064, respectively). Although the specificity, accuracy, and AUC for protocol C were better than those for protocol B, no significant difference was observed (0.25, 0.25, and 0.34, respectively).

**Fig 2:**
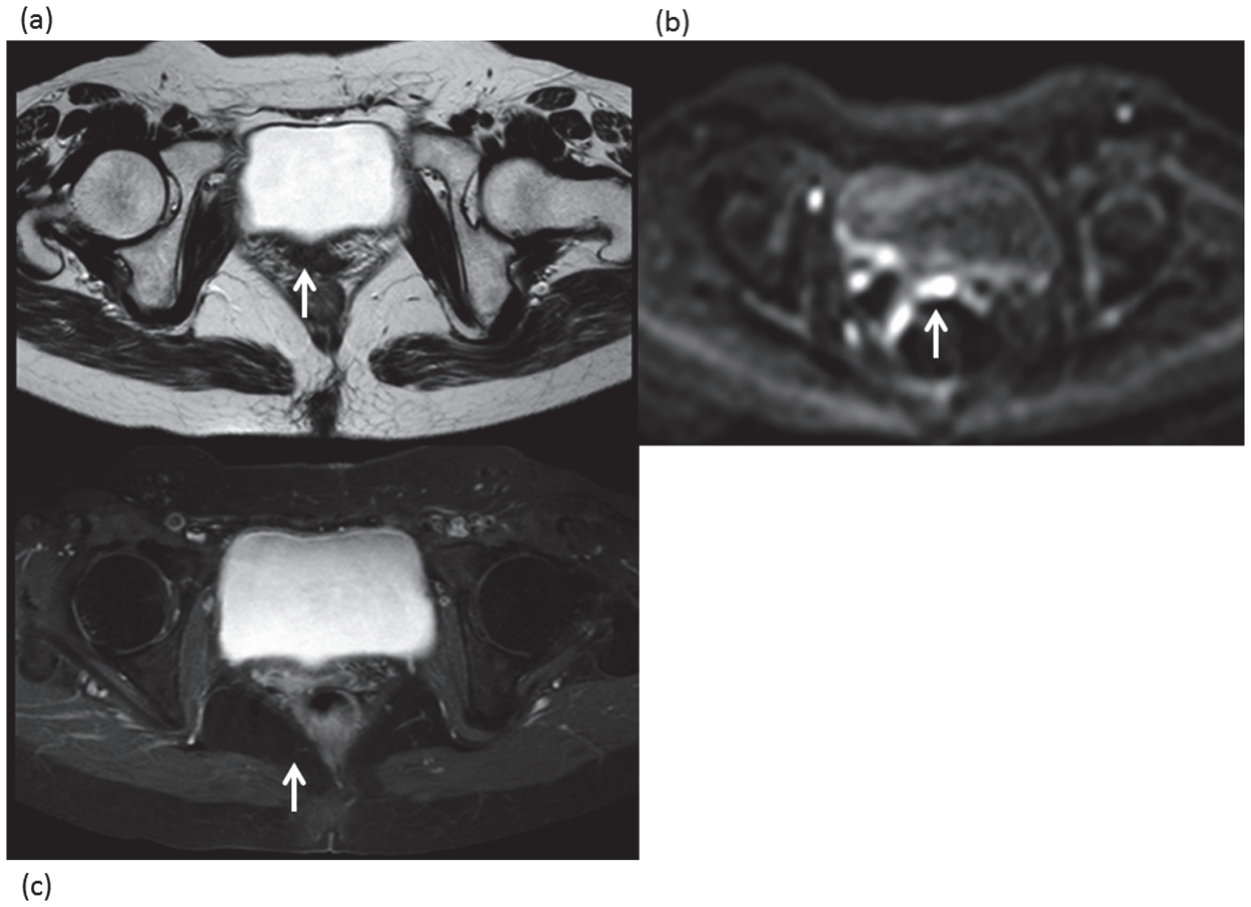
A 54-year-old woman with a locally recurrent tumor 10 months after surgery for uterine cervical cancer. (a) Axial T2-weighted MRI shows a small, slightly hyperintense area in the vaginal vault (arrow). This equivocal finding for local recurrence was assigned a score of 3 for protocol A. (b) Axial DWI shows a focal hyperintense area in the posterior urinary bladder (arrow), corresponding to the area seen in (a), suggesting local recurrence. Protocol B was assigned a score of 5 for local recurrence. (c) Axial contrast-enhanced MRI shows abnormal enhancement as the same site (arrow), suggesting local recurrence. The score assigned for protocol C was 5. Biopsy confirmed the presence of tumor recurrence.

**Fig 3:**
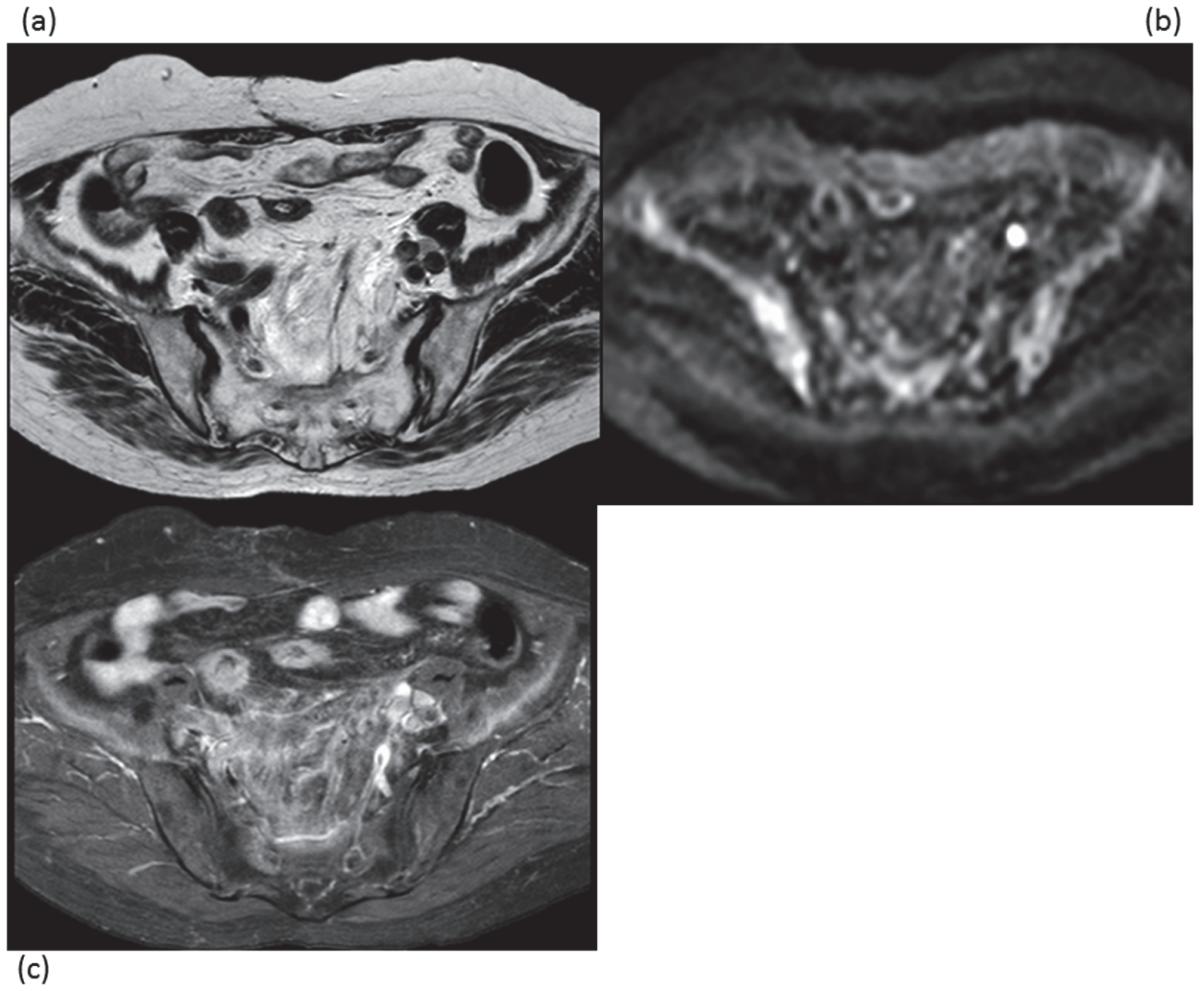
A 65-year-old woman with intrapelvic lymph node recurrence 12 months after surgery for endometrial cancer. (a) Axial T2-weighted MRI shows a 9-mm left external iliac LN (arrow). This equivocal finding for lymph node recurrence was assigned a score of 3 for protocol A. (b) Axial DWI shows a focal hyperintense spot in the left external iliac area (arrow), corresponding to the node seen in (a), being suspicious for lymph node recurrence. The score assigned for protocol B was 4. (c) Axial contrast-enhanced MRI shows slight ring-like enhancement of the same node (arrow), suggesting lymph node recurrence. The score assigned for protocol C was 4. Follow-up MRI showed an increase in the size of this node, confirming lymph node recurrence.

Three representative cases are shown in Figs. 2–3.

## Discussion

In the present series, MRI with a combination of DWI and T2WI showed a significantly better diagnostic performance relative to T2WI alone, and a performance as high as that of contrast-enhanced imaging for assessment of intrapelvic recurrence of gynecological malignant tumors. Although DWI showed slightly lower sensitivity for detection of peritoneal dissemination and slightly lower specificity for evaluation of local recurrence in comparison with contrast-enhanced imaging, the differences between the two methods were not significant.

DWI allows excellent delineation of malignant tumors because of the generally suppressed background noise. However, we consider it necessary to refer to other imaging sequences for sufficient identification of lesion boundaries. We found that the combination of DWI and conventional non-enhanced MRI identified additional sites of pelvic tumors and improved the degree of confidence for image interpretation. Additional advantages of DWI include its completely non-invasive nature and cost effectiveness. DWI does not involve radiation exposure, or oral or intravenous administration of contrast material, and is not uncomfortable for the patient. DWI can be easily added to MR study protocols and no additional time for injection of contrast material is required. In patients with gynecological malignancies, DWI can play an important role in the detection of localized tumor recurrence within the pelvis as well as disseminated peritoneal recurrence.

To our knowledge, only one previous study has demonstrated the usefulness of fused DWI for assessment of intrapelvic recurrence of gynecological malignancy in comparison with dynamic contrast-enhanced MRI. Nishie et al. [10] reported that high signal intensity on high-*b*-value DWIs was an indicator of local recurrence of pelvic malignancies (mostly uterine cervical cancers) in a study involving 28 patients. They considered that the diagnostic performance of fused T2WI and DWI (sensitivity 94%, specificity 72%) was comparable to that of DCE-MRI (sensitivity 96%, specificity 58%).

Diagnosis of local recurrence at the vaginal vault after hysterectomy for uterine malignancy using CT and FDG-PET/CT is often difficult due to limited spatial and contrast resolution. dynamic contrast-enhanced MRI has been shown to be useful for detection of local recurrence at the vaginal vault after hysterectomy. Kinkel et al. [2] found that dynamic contrast-enhanced MRI with analysis of signal intensity-time curves improved the ability of MRI to detect tumor recurrence following radiotherapy in 15 confirmed cases of recurrent uterine cervical cancer. They compared dynamic contrast-enhanced MRI with standard T2WI, and found that the specificity and accuracy increased from 22% and 68% to 67% and 83%, respectively. In our present series, the ability of DWI to detect local recurrence at the vaginal vault after hysterectomy was equivalent to that of contrast-enhanced imaging, as Nishie et al. had reported [10]. Although 20 patients with uterine cancers had local recurrence at the vagina (vaginal vault in 12 and one-third in 8) in our series, Blecharz et al. analyzed the characteristics of vaginal and pelvic recurrences in 106 patients with stage I and II endometrial carcinoma and demonstrated that 17 (16%) patients had vaginal vault recurrences, 30 (28.3%) had lower one-third vaginal recurrences, and 59 (55.7%) had pelvic recurrences [11].

The peritoneal cavity is a common site for metastatic spread of gynecological malignancies, especially in patients with ovarian cancer. In such patients, MRI is very useful for follow-up of the treatment response and for detection of recurrent disease. It is important to realize that second-look surgery is no longer routine, and that imaging diagnosis of recurrence may obviate a second-look laparotomy, since secondary cytoreduction is only justified if resection is considered likely to leave no residual tumor. dynamic contrast-enhanced MRI is comparable (sensitivity 90%, specificity 88%, and accuracy 89%) to laparotomy (sensitivity 88%, specificity 100%, accuracy 89%) but superior to serum CA-125 analysis (sensitivity 65%, specificity 88%, and accuracy 67%) for detection of residual or recurrent peritoneal and serosal implants in women who have been treated for ovarian cancer [4]. DWI can also clearly discriminate the abnormal signal intensity of peritoneal dissemination from signals arising from surrounding organs such as the bowel. In our series, the ability of DWI to detect intrapelvic peritoneal dissemination was comparable to that of contrast-enhanced imaging. Fujii et al. [12] also showed that DWI using 1.5 Tesla scanner was highly sensitive (90%) and specific (95.5%) for evaluation of peritoneal dissemination in the initial staging of ovarian cancer (n = 26).

There were several limitations to this study. First, it was retrospective in design with a relatively small sample size. Further prospective and larger studies will therefore be needed. Second, it was a retrospective analysis of patients presenting over a five-year period. The changes in MRI parameters that occurred over the course of this period may have influenced the performance of the readers. The part of the patients have been scanned with 1.5, and the other part with 3.0 Tesla scanners. DCE-MRI was performed in half of the patients, and the remaining half underwent one-shot delayed contrast-enhanced MRI. Therefore, the results of protocol C may have been underestimated. Apparent diffusion coefficient measurements were not performed for DWI because this was beyond the scope of our retrospective study. Third, the ideal gold standard for any analysis would be histological confirmation of the findings. However, clinical follow-up is also a valid approach for assessment of diagnostic accuracy and response to therapy, and it would have been unethical to use invasive procedures for investigating all lesions detected by imaging. Positive findings are easy to confirm, but negative findings only reflect the fact that no positive findings are acquired during the follow-up period, making it uncertain whether the findings are indeed truly negative. Therefore, sensitivity in this series may have been overestimated. Fourth, although it would have been interesting to compare MRI and FDG-PET/CT for assessment of intrapelvic recurrence of gynecological malignancies, such analysis could not be performed in our series because patients who had undergone both MRI and FDG-PET/CT examinations were limited. In another study involving 30 patients with gynecological malignancy, we have also reported that MRI including DWI and CEI showed better sensitivity than FDG-PET/CT for depicting local recurrence (88% vs. 63%), the same sensitivity for detection of intrapelvic bony metastasis (67%), and worse sensitivity for evaluation of intrapelvic lymph node recurrence (63% vs 88%) and peritoneal dissemination (60% vs. 80%) [13]. Satoh et al. demonstrated that diffusion-weighted MRI without contrast enhanced imaging and FDG-PET/CT showed the almost same high diagnostic performance of peritoneal dissemination in more than 100 patients [14]. Fifth, DWI has several pitfalls including low signal-to-noise ratio, susceptibility artifacts, and T2 shine-through. Several false negative findings (e.g., well-differentiated adenocarcinomas or ovarian cancers with large cystic components, or poorly differentiated necrotic tumors) and false positive cases (e.g.,blood, fat, abscesses, reactive lymph nodes, and melanin) are often observed [15].

## Conclusion

MRI with a combination of DWI and T2WI shows a high diagnostic performance and is comparable to contrast-enhanced imaging for assessment of intrapelvic recurrence of gynecological malignant tumors. DWI without contrast material may be a useful modality.
